# Factors Influencing the Duration of Maintenance Therapy in Metastatic Colorectal Cancer

**DOI:** 10.3390/cancers17010088

**Published:** 2024-12-30

**Authors:** Théo Fourrier, Caroline Truntzer, Morgane Peroz, Valentin Derangère, Julie Vincent, Leila Bengrine-Lefèvre, Audrey Hennequin, Rémi Palmier, David Orry, Thomas Rabel, François Ghiringhelli

**Affiliations:** 1 Cancer Biology Transfer Platform, Georges François Leclerc Cancer Center, UNICANCER, 21000 Dijon, France; 2 Department of Medical Oncology, Georges François Leclerc Cancer Center, UNICANCER, 21000 Dijon, France; 3 INSERM UMR1231 Research Center, University of Burgundy, 21000 Dijon, France; 4 Department of Surgical Oncology, Georges François Leclerc Cancer Center, UNICANCER, 21000 Dijon, France

**Keywords:** metastatic colorectal cancer, chemotherapy, targeted therapies, induction treatment, maintenance treatment

## Abstract

Background/Objectives: Metastatic colorectal cancer (mCRC) is mainly treated with 5-Fluoro-Uracil (5-FU), Oxaliplatin and Irinotecan chemotherapies and anti-Epidermal Growth Factor Receptor (EGFR) or anti-Vascular Endothelial Growth Factor (VEGF) targeted therapies. Due to chemotherapy-related toxicity, patients receive induction treatment to achieve tumour response followed by maintenance therapy with less cytotoxic molecules or a chemotherapy-free interval to reduce chemotherapy-related toxicity. In this study, the aim was to determine the patient, cancer and treatment factors that influence the duration of maintenance therapy (DMT). Methods: We collected retrospective data on a cohort of 133 patients treated at the Centre Georges François Leclerc (CGFL) cancer centre in Dijon between March 2014 and June 2022. Patients had unresectable or potentially resectable diseases. They received first-line induction treatment with chemotherapy and/or targeted therapy and maintenance treatment, defined as the interruption of at least one chemotherapy agent. Results: In the multivariate analysis, age (HR: 1.02, 95% CI 1.00–1.04, *p* = 0.031), N2 nodal status (HR: 1.78, 95% CI 1.09–2.89, *p* = 0.021) and the presence of peritoneal metastases (HR: 2.05, 95% CI 1.25–3.36, *p* = 0.004), as well as baseline carcino-embryonic antigen (CEA) level (HR: 1.10, 95% CI 1.00–1.20, *p* = 0.052), were significantly associated to poor DMT. Local treatment of liver metastases also significantly reduced the DMT (HR: 0.49, 95% CI 0.28–0.86, *p* = 0.013). In our cohort, induction triplet chemotherapy significantly increased the CEA delta (70% vs. 44%, *p* = 0.047) compared to doublet chemotherapy and led to a higher rate of liver surgery (40% vs. 21%, *p* = 0.014) and a trend for a higher rate of local treatment of metastases (62% vs. 45%, *p* = 0.059). Conclusions: Duration of maintenance therapy is determined by the initial patient and colorectal cancer characteristics. However, it is significantly increased by local treatment of liver metastases. By reducing the tumour burden, a triplet induction chemotherapy regimen increases the rate of liver metastase resection.

## Simple Summary

Due to chemotherapy-related toxicity, metastatic colorectal cancer is often treated initially by induction therapy followed by maintenance therapy with less cytotoxic molecules or a chemotherapy-free interval. The maintenance therapy period is a time with theoretically fewer cancer-related symptoms and less treatment-related toxicity. For patients with unresectable metastatic colorectal cancer, one of the aims of palliative treatment is to maximise the duration of maintenance therapy. In this study, we aimed to determine the factors that influence the duration of maintenance therapy. In our cohort of patients, patient and colorectal cancer characteristics but also primary tumour resection and local treatment of liver and lung metastases determined the duration of maintenance therapy. The induction chemotherapy regimen influenced the access to local treatment of metastases, whereas the maintenance chemotherapy regimen did not modify survival.

## 1. Introduction

Colorectal cancer (CRC) is a significant global health issue, with nearly 2 million new cases and 1 million deaths annually. It is the third most common cancer in both sexes [1]. In France, approximately 47,000 new cases and over 17,000 deaths were reported last year, according to the National Cancer Institute (INCa).

CRC is classified into localised and metastatic diseases for prognosis and treatment. While localised CRC has a 5-year survival rate of approximately 90%, metastatic CRC (mCRC) has a survival rate below 20% [2]. Around 15–30% of patients are diagnosed with synchronous metastases, and 20–50% of initially localised cases progress to metachronous metastases [3].

Over three decades, mCRC treatment has advanced significantly. 5-Fluoro-Uracil (5-FU), initially administered as a bolus and later as a continuous infusion with Leucovorin (LV5FU2), marked a key milestone in improving overall survival (OS) [4]. The early 2000s saw the introduction of Irinotecan and Oxaliplatin, enhancing objective response rates (ORR) and OS in combination with 5-FU, achieving a median OS of 16–17 months [5,6]. Doublet chemotherapy regimens such as FOLFIRI (5-FU and Irinotecan) and FOLFOX (5-FU and Oxaliplatin) became standard treatments for mCRC.

Targeted therapies, including anti-Epidermal Growth Factor Receptor (EGFR) monoclonal antibodies (Cetuximab, Panitumumab), showed efficacy in later lines versus best supportive care (BSC) [7,8]. These therapies were later combined with doublet regimens, increasing median OS to 24 months in KRAS wild-type (KRAS^WT^) patients, as seen in the PRIME and CRYSTAL studies [9,10,11].

Anti-Vascular Endothelial Growth Factor (VEGF) agents (Bevacizumab, Aflibercept) normalise tumour vasculature and improve tumour perfusion, enabling their potential as chemosensitisers [12]. Combined with chemotherapy, these treatments increased median OS to 20–21 months, irrespective of RAS gene status [13,14,15].

For KRAS^WT^ patients, the FIRE-3 and PEAK studies showed a significant OS advantage with anti-EGFR over anti-VEGF therapy, achieving a median OS of 28 months when combined with doublet regimens [16,17]. Retrospective analyses identified the primary tumour location (PTL) as a prognostic factor and indicated that left-sided mCRC patients with KRAS^WT^ benefited most from anti-EGFR therapy [18,19].

The Gruppo Oncologico Nord Ovest (GONO) group showed that the triplet chemotherapy regimen FOLFIRINOX (5-FU, Irinotecan, Oxaliplatin) improved ORR and OS compared to the FOLFIRI doublet regimen [20]. Following these results, targeted therapies combined with triplet chemotherapy demonstrated further benefits. The TRIBE study revealed significant improvements in progression-free survival (PFS) and OS (30 months) with FOLFIRINOX and Bevacizumab compared to FOLFIRI with Bevacizumab [21,22]. However, the TRIPLETE study (2022) found no ORR or PFS improvement when Panitumumab was added to triplet chemotherapy, and increased toxicity was observed [23].

Patients with potentially resectable mCRC benefit from higher response rates, allowing more access to curative surgeries. In the CELIM trial, Cetuximab combined with FOLFOX or FOLFIRI achieved high ORR and R0 resection rates. Similarly, the OLIVIA trial showed favourable outcomes with Bevacizumab and FOLFIRINOX or FOLFOX [24,25]. Advances in liver surgery techniques (extended hepatectomy, portal embolisation [26], two-stage hepatectomy [27], thermal ablation [28]) have increased surgical eligibility, improving patient prognosis [2].

Primary tumour resection (PTR) has shown OS benefits in retrospective studies in clinically selected patients with initially unresectable mCRC [29]. However, the CAIRO4 prospective study found no survival benefit from upfront PTR combined with systemic therapy in unselected patients [30].

Medical and surgical management of mCRC aims to improve both OS and quality of life. Chemotherapy regimens, while effective, are highly toxic. Common side effects include fatigue, haematological toxicity, hand-foot syndrome, mucositis (5-FU), peripheral neuropathy (Oxaliplatin), and severe diarrhoea (Irinotecan) [5,6]. Gene polymorphisms in dihydropyrimidine dehydrogenase (DPYD) and uridine diphosphate glucoronosyltransferase (UGT1A1), enzymes involved in 5-FU and Irinotecan metabolism, influence the severity of these toxicities [31,32,33].

Targeted therapies also have adverse effects: anti-EGFR therapies cause skin reactions and diarrhoea [10,11], while anti-VEGF therapies may lead to hypertension, proteinuria, and thromboembolic or hemorrhagic events [14,15].

These toxicities often result in treatment interruption. In the OPTIMOX1 study, C. Tournigand et al. introduced the treatment paradigm of induction therapy with doublet chemotherapy (FOLFOX7) for 6 cycles to induce a tumour response, followed by maintenance therapy with single chemotherapy (LV5FU) for 12 cycles to maintain the tumour response and reduce treatment toxicity. This strategy did not alter PFS or OS, while grade 3–4 toxicities, including peripheral neuropathy, were reduced in the maintenance therapy group [34].

The COIN trial investigated the potential feasibility of intermittent chemotherapy during which mCRC patients had six cycles of FOLFOX followed by a chemotherapy-free interval (CFI) until disease progression, at which point the treatment was restarted. The trial did not reach non-inferiority but showed a significant reduction of grade 3–4 toxicities, including hand-foot syndrome and peripheral neuropathy, and an improvement in patient-reported quality of life [35].

The OPTIMOX2 study demonstrated an advantage for maintenance therapy (LV5FU) compared to intermittent chemotherapy with CFI on PFS and duration of disease control (DDC) after six cycles of induction chemotherapy by mFOLFOX7 [36], thus raising caution regarding the usage of the intermittent chemotherapy strategy.

In the FOCUS4-N trial, after eight cycles of induction chemotherapy by FOLFOX or FOLFIRI, Capecitabine as maintenance therapy showed an improvement of PFS compared to no chemotherapy until disease progression and restart of induction chemotherapy. However, OS was not statistically different in the two groups and patients under intermittent chemotherapy had fewer treatment-related toxicities [37].

Several additional studies have investigated targeted therapies as maintenance therapy. Anti-EGFR agents (Cetuximab and Panitumumab) improved PFS as maintenance therapy alone or in association with LV5FU in the TIME and PANAMA trials after induction chemotherapy by FOLFIRI or FOLFOX [38,39]. Anti-VEGF agent Bevacizumab also improved PFS in association with Capecitabine as maintenance therapy after six cycles of CAPOX (Capecitabine and Oxaliplatin)-Bevacizumab in the CAIRO3 study [40]. However, the PRODIGE 9 study results showed that Bevacizumab maintenance therapy alone did not improve PFS and OS compared to no maintenance treatment after 12 cycles of induction treatment by FOLFIRI-Bevacizumab [41].

The principal aim of palliative cancer treatment is to maximise the duration of life of patients without cancer-related symptoms and minimise treatment-induced toxicity. The duration of maintenance therapy, defined as the time interval between the last administration of the induction therapy and disease progression, is a time interval during which patients have theoretically fewer cancer-related symptoms and fewer toxic effects.

Our aim was to carry out a retrospective review in order to identify mCRC patient and disease characteristics that influence treatment choice for induction and maintenance therapy and the impact of these characteristics and treatments on the duration of maintenance therapy (DMT).

## 2. Materials and Methods

### 2.1. Patient Selection

Patients with histologically proven metastatic colorectal cancer treated between March 2014 and June 2022 at the Centre Georges François Leclerc (CGFL) cancer centre in Dijon were included. All incident patients who received at least one cycle of chemotherapy during this period were included in the study. Additional inclusion criteria were the presence of synchronous or metachronous metastases with unresectable or potentially resectable disease, the administration of first-line induction treatment with bi or trichemotherapy with or without targeted therapy and a maintenance treatment defined as the interruption of at least one chemotherapy agent. Exclusion criteria were the presence of another active cancer, the presence of a resectable metastatic cancer, and treatment with monochemotherapy. A total of 133 patients met these inclusion/exclusion criteria. The study was conducted in accordance with the Declaration of Helsinki, revised in 2013.

### 2.2. Data Collection

Clinical data was collected retrospectively from patients’ medical records.

Demographics and Performance Status

Age, gender, and Eastern Cooperative Oncology Group (ECOG) performance status (PS).

Pharmacogenetic Mutations

Dihydropyrimidine Dehydrogenase (DPYD, is classified as proficient, intermediate, or deficient based on gene polymorphisms. Uridine Diphosphate Glucuronosyltransferase 1A1 (UGT1A1) promoter region categorised as Wild Type (WT) (UGT1A1*1/1), Heterozygous (UGT1A1*1/*28) or Homologous (UGT1A1*28/*28).

Colorectal Cancer Clinical Characteristics

Primary Tumor and Disease Features: primary tumour location, presence of intestinal obstruction, Tumor-Node-Metastasis (TNM) stage, timing of metastases: metachronous or synchronous. Baseline Biomarkers: baseline carcinoembryonic antigen (CEA) levels.

Colorectal Cancer Histological and Molecular Characteristics

Histological subtype, KRAS, NRAS, and BRAF mutations, Mismatch Repair (MMR) and Microsatellite Instability (MSI) status.

Treatment Characteristics

Induction Therapy: chemotherapy regimen, targeted therapy regimen, therapy duration, radiologic and biological responses, including nadir CEA and CEA delta. Maintenance Therapy: chemotherapy regimen, targeted therapy regimen. Local Treatments: primary tumour resection, surgical and non-surgical local metastasis treatments (e.g., radiotherapy, thermal ablation). Dose Intensities and Total Cumulated Doses:5-FU and Irinotecan induction dose intensities and total cumulated doses

### 2.3. Statistical Analysis

We compared the characteristics of mCRC patients receiving either triplet or doublet regimens as induction chemotherapy and doublet, single or no maintenance chemotherapy.

The primary endpoint was to determine the factors influencing the duration of maintenance therapy, which is defined as the time interval between the last administration of the induction therapy and disease progression.

The secondary endpoint was to determine the factors influencing overall survival, which was defined as the time interval between the beginning of induction therapy and patient death or the date of the last follow-up. All patients without an event at the end of follow-up were censored.

We also analysed the treatment characteristics and survival data of pharmacogenetic subpopulations with polymorphisms of DPYD and/or UGT1A1 genes.

Follow-up data in medical records was reviewed until the patient death or the date of last follow-up prior to March 2024. Survival data was censored at the date of the last follow-up. Comparative statistical analyses were performed with the Wilcoxon–Mann Whitney test for quantitative variables and Pearson’s Chi-squared test or Fisher’s exact test for qualitative variables. Univariate and multivariate Cox regression models were applied to determine the factors influencing the duration of maintenance therapy or overall survival. Survival curves are represented with the Kaplan–Meier method and compared using the log-rank test. The significance threshold was pre-defined at 5% (*p* < 0.05). Statistical analysis was performed with R^®^ version 4.2.2, Vienna, Austria, and figures were generated using Graph Pad Prism^®^ version 9, La Jolla, California, United States.

## 3. Results

### 3.1. Patient Characteristics

The mean age at diagnosis was 65 years, gender was predominantly male (59%), and almost 90% of patients had a performance status (PS) of 0–1, and 11% had a PS2 (see Table 1). Of the 104 patients who received genetic characterisation, 9 patients (9%) were intermediate or deficient for DPYD, 42 (39%) patients had a heterozygous mutation, and 13 (12%) had a homologous mutation of the promoter of UGT1A1.

As for the metastatic colorectal cancer characteristics, the primary tumour location was left-sided in 37%, right-sided in 27% and rectal in 35% of cases. Tumours were mainly T3 (67%) with N1 (43%) or N2 (39%) nodal status. A total of 47% of mCRC patients had one metastatic site, 25% of patients had two metastatic sites, and 28% of patients had peritoneal metastases. Most patients had synchronous metastatic disease (77%), and the mean baseline CEA was 418 U/L. A total of 29 (22%) patients had an intestinal occlusion or sub-occlusion.

The histological subtype was largely Lieberkuhnian adenocarcinoma (95%), and KRAS, NRAS and BRAF genes were mutated in 58%, 4% and 8% of cases, respectively. Three patients had deficient MMR/MSI status.

Referring to treatment characteristics, 45% of patients received a triplet chemotherapy regimen (FOLFIRINOX) as induction therapy, while 55% of patients received a doublet chemotherapy with FOLFOX (50%) or FOLFIRI (5%). Induction chemotherapy duration was 3.5 months on average and was associated with an anti-VEGF agent in 55% of cases or an anti-EGFR agent in 22% of cases. Induction treatment led to partial response (PR), which is a vast majority of cases (76%), and a mean nadir CEA of 29 U/L with a mean CEA delta of 80%.

Maintenance chemotherapy was a single chemotherapy (LV5FU or Capecitabine) in 53% of cases. Doublet chemotherapy (FOLFOX, FOLFIRI or CAPOX) was administered in 25% of cases following induction triplet chemotherapy. A total of 22% of patients had a chemotherapy-free interval. An anti-VEGF agent was administered in 49% of cases, and an anti-EGFR agent in 17% of cases.

Across the cohort of mCRC patients, 72% of patients underwent primary tumour resection, of which 68% had synchronous metastatic disease. A total of 33% of patients had a local treatment of liver metastases, including 39 hepatic surgeries. A total of 17 patients (13%) had local treatment for lung metastases, and 13 patients (10%) had peritoneal surgery.

The survival data showed a median duration of maintenance therapy was 13.9 months (95% CI 11.2–15.6), median PFS was 17.9 months (95% CI 14.9–19.4), and median OS was 54.9 months (95% CI 39.4–66.5).

### 3.2. Comparison of the Characteristics of Patients Treated by Triplet or Doublet Induction Chemotherapy Regimen

Patients treated with induction triplet chemotherapy regimen (FOLFIRINOX) were significantly younger (mean age: 61 years vs. 68 years, *p* < 0.001) and in better general condition (PS 0–1: 97% vs. 82%, *p* = 0.006) than patients treated by induction doublet chemotherapy regimen (FOLFOX or FOLFIRI) (see Table 2).

Patients who received triplet chemotherapy had a higher proportion of rectal tumours (47% vs. 25%, *p* = 0.038), whereas patients receiving doublet chemotherapy had a high proportion of left-sided colon tumours (44% vs. 30%, *p* = 0.038).

There were no significant differences in the TNM stage or histological and biomolecular characteristics. However, patients treated with triplet chemotherapy had a higher rate of synchronous metastases (87% vs. 68%, *p* = 0.014) and a higher mean baseline CEA (683 U/L vs. 185 U/L, *p* = 0.003).

Both triplet and doublet induction chemotherapy were associated with anti-VEGF targeted therapy in the majority of cases (57% and 54%, respectively). Doublet chemotherapy was administered without targeted therapy more often than was seen in triplet chemotherapy (25% vs. 12%, *p* = 0.032). Radiological objective response rates were similar across the two groups (83% in the triplet chemotherapy and 77% in the doublet chemotherapy group, *p* = 0.4). However, the CEA delta was significantly higher in the triplet chemotherapy group (70% vs. 44%, *p* = 0.047).

Induction triplet chemotherapy was associated with 53% of doublet maintenance chemotherapy, 19% of single maintenance chemotherapy and a chemotherapy-free interval in 17% of cases. This compares to induction doublet chemotherapy being associated with 73% single-agent maintenance chemotherapy and 27% of patients having a chemotherapy-free interval as maintenance (*p* < 0.001).

Induction triplet chemotherapy led to a higher rate of liver surgery than doublet chemotherapy (40% vs. 21%, *p* = 0.014) and a trend for a higher rate of local treatment of metastases (62% vs. 45%, *p* = 0.059). Primary tumour resection rates were similar across the two groups (70% in the triplet chemotherapy and 74% in the doublet chemotherapy group, *p* = 0.6).

**Figure 1:**
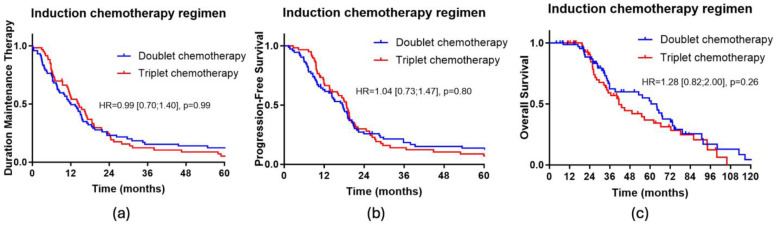
(**a**) Kaplan-Meier curve of the DMT depending on the induction chemotherapy regimen; (**b**) Kaplan-Meier curve of PFS depending on the induction chemotherapy regimen; (**c**) Kaplan-Meier curve of OS depending on the induction chemotherapy regimen.

Regarding survival characteristics, there was no significant difference in the duration of maintenance therapy, progression-free survival or overall survival across the induction triplet or doublet chemotherapy groups (see Figure 1).

### 3.3. Comparison of the Characteristics of Patients Treated by Doublet, Single or No Maintenance Chemotherapy Regimen

Among the initial 133 patients, 125 patients received a maintenance therapy or a chemotherapy break interval. Eight patients progressed during induction therapy.

Patients receiving maintenance doublet chemotherapy had a significantly younger mean age than patients receiving single or no chemotherapy (60 years vs. 70 and 69 years, *p* = 0.025). They also had a higher rate of synchronous metastases (90% vs. 76% and 61%, *p* = 0.029) and a trend for a higher mean baseline (766 vs. 290 and 485, *p* = 0.068) (see Table S1 in Supplementary Materials).

There were no other significant clinical differences between patient and colorectal cancer, as well as histopathological or biomolecular characteristics.

Maintenance doublet chemotherapy always followed induction triplet chemotherapy. Maintenance single chemotherapy was preceded by triplet chemotherapy in 26% of cases and doublet chemotherapy in 74% of cases. Chemotherapy-free interval was accorded in 36% of cases after triplet chemotherapy and 64% of cases after doublet chemotherapy.

Induction therapy radiological response rates and biological response were similar in the different maintenance chemotherapy groups.

Anti-VEGF agents were administered with doublet and single maintenance chemotherapy in 58% and 66% of cases, while anti-EGFR agents were administered in 19% and 20% of cases. Chemotherapy breaks were often associated with targeted therapy breaks (89%).

Patients receiving maintenance doublet chemotherapy had a higher rate of local treatment of liver metastases than patients receiving single chemotherapy or no chemotherapy (48% vs. 24% and 46% respectively, *p* = 0.025). Patients with single-maintenance chemotherapy had a significantly lower rate of any local treatment of metastases than patients receiving doublet chemotherapy or no chemotherapy (44% vs. 65% vs. 75%, respectively, *p* < 0.012).

**Figure 2:**
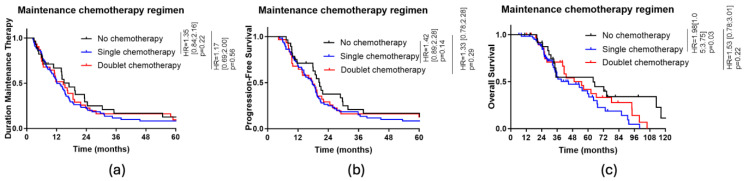
(**a**) Kaplan-Meier curve of the DMT depending on the maintenance chemotherapy regimen; (**b**) Kaplan-Meier curve of PFS depending on the maintenance chemotherapy regimen; (**c**) Kaplan-Meier curve of PFS depending on the maintenance chemotherapy regimen.

Overall, there were no differences in the duration of maintenance therapy, progression-free survival or overall survival between the three groups. Surprisingly, a significant difference in OS was observed in favour of no chemotherapy vs. single chemotherapy as a maintenance treatment (HR: 1.98, 95% CI 1.05–3.75, *p* = 0.03) (see Figure 2).

### 3.4. Determination of the Characteristics Influencing the Duration of Maintenance Therapy

#### 3.4.1. Univariate Analysis

Patient characteristics data analysis showed that the age at diagnosis (HR: 1.01, 95% CI: 1.00–1.03, *p* = 0.072), performance status score of 1 (HR: 1.48, 95% CI 1.02–2.14, *p* = 0.039) and 2 (HR: 2.54, 95% CI: 1.36–4.74, *p* = 0.004) reduced the duration of maintenance therapy (DMT) (see Table 3).

Among the mCRC clinical characteristics, the N2 stage (HR: 1.91, 95% CI 1.24–2.95, *p* = 0.003), the number and location of the metastatic sites M1b (HR: 1.71, 95% CI 1.11–2.64, *p* = 0.015) and M1c (HR: 1.84, 95% CI 1.20–2.83, *p* = 0.005) were associated with a reduction in DMT. The synchronous occurrence of metastases (HR: 1.54, 95% CI 1.02–2.35, *p* = 0.042) and baseline CEA (HR: 1.09, 95% CI 1.01–1.18, *p* = 0.026) were also associated with a shorter DMT.

mCRC histological and molecular characteristics did not significantly influence the DMT.

Analysis of treatment characteristics showed no significant difference between induction doublet or triplet chemotherapy or targeted therapies with respect to DMT. High nadir CEA was associated with a reduction in DMT (HR: 1.21, 95% CI 1.08–1.35, *p* = 0.001), and ORR had a trend for longer DMT (HR: 0.70, 95% CI 0.45–1.10, *p* = 0.12).

Maintenance of doublet, single or no chemotherapy regime did not significantly influence the DMT. Surprisingly, anti-VEGF maintenance targeted therapies were associated with a shorter DMT (HR: 1.66, 95% CI 1.09–2.53, *p* = 0.018).

Surgical treatments, including primary tumour resection and local treatment of liver and lung metastases, considerably increased the DMT (HR: 0.45, 95% CI 0.30–0.68, *p* < 0.001, HR: 0.61, 95% CI 0.41–0.90, *p* = 0.012 and HR: 0.40 95% CI 0.38–0.77, *p* = 0.005 respectively).

#### 3.4.2. Multivariate Analysis

In the multivariate analysis, age remained a statistically significant determinant of DMT (HR: 1.02, 95% CI 1.00–1.04, *p* = 0.031). N2 (HR: 1.78, 95% CI 1.09–2.89, *p* = 0.021), M1c status (HR: 2.05, 95% CI 1.25–3.36, *p* = 0.004) as well as baseline CEA (HR: 1.10, 95% CI 1.00–1.20, *p* = 0.052) also remained significantly associated to poor DMT (see Table 4).

Among the treatment characteristics, local treatment of liver metastases significantly increased the DMT in the multivariate analysis (HR: 0.48, 95% CI 0.26–0.88, *p* = 0.017).

### 3.5. Determination of the Characteristics Influencing the Overall Survival

#### 3.5.1. Univariate Analysis

Among patient characteristics, performance status of 2 was a severe prognosis factor for OS (HR: 3.13, 95% CI 1.26–7.79, *p* = 0.014) (see Table S2 in Supplementary Materials).

Of colorectal cancer characteristics, the presence of peritoneal metastases (M1c) had a non-significant trend on OS (HR: 1.68, 95% CI 0.97–2.92, *p* = 0.065).

The three patients with dMMR/MSI status had a favourable outcome in OS (HR: 0.14, 95% CI 0.02–1.01, *p* = 0.052).

The treatment characteristics analysis showed that single maintenance chemotherapy was associated with a poorer outcome (HR: 1.97, 95% CI 1.04–3.72, *p* = 0.037), whereas primary tumour resection (HR: 0.52, 95% CI 0.28–0.94, *p* = 0.032), as well as local treatment of liver metastases (HR: 0.60, 95% CI 0.38–0.97, *p* = 0.036), were favourable for OS. The 13 patients who underwent peritoneum metastase surgery had a worse outcome (HR: 3.46, 95% CI 1.75–6.85, *p* < 0.001).

#### 3.5.2. Multivariate Analysis

Primary tumour resection remained significantly associated with a favourable outcome (HR: 0.48, 95% CI 0.23–0.99, *p* = 0.045), while peritoneum metastases surgery remained associated with a worse prognosis (HR: 3.86, 95% CI 1.71–8.68, *p* = 0.001) (see Table S3 in Supplementary Materials).

### 3.6. Comparison of the Characteristics of Pharmacogenetic Subpopulations Based on DPYD and/or UGT1A1 Gene Polymorphism

Respectively, 104 and 117 patients benefited from the genetic characterisation of DPYD and UGT1A1 polymorphisms.

Concerning the DPYD gene, 95 patients were proficient, 8 were intermediate, and 1 patient was deficient. Intermediate and deficient groups were merged for analysis.

Comparing the DPYD proficient and DPYD intermediate/deficient patients, there were no significant differences in induction 5-FU dose intensity (94% vs. 89%, *p* = 0.5) or 5-FU cumulated dose (114,925 vs. 126,456 mg, *p* > 0.9) (see Table 5). FOLFIRINOX induction chemotherapy regime was received in 51% of cases for DPYD proficient patients vs. 33% for DPYD intermediate/deficient patients, *p* = 0.3. Radiological response rates were similar across the two groups.

**Figure 3:**
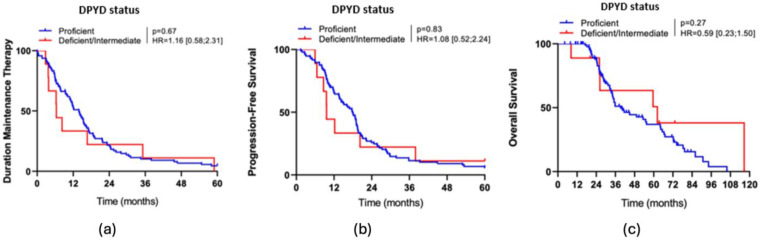
(**a**) Kaplan-Meier curve of the DMT depending on DPYD status; (**b**) Kaplan-Meier curve of the PFS depending on DPYD status; (**c**) Kaplan-Meier curve of the OS depending on DPYD status.

Patients with intermediate or deficient DPYD did not have a statistically significant reduction in DMT, PFS or OS (see Figure 3).

Concerning the UGT1A1 gene, 53 patients were WT, 41 patients were heterozygous, and 13 patients had homologous mutations of the promoter region.

Comparing the three subpopulations, there was a significant difference in induction Irinotecan dose intensity (86% vs. 83% vs. 37% respectively, *p* = 0.025) and a non-significant trend for the difference in Irinotecan cumulated dose (2.932 mg vs. 3.290 mg vs. 1.556 mg respectively, *p* = 0.13) (see Table 6). Patients with UGT1A1 homologous mutations had a higher rate of FOLFOX regimen as induction chemotherapy (77% vs. 40% and 27% in the WT and heterozygous mutation populations, respectively, *p* = 0.059). Overall response rates were similar between the three groups.

**Figure 4:**
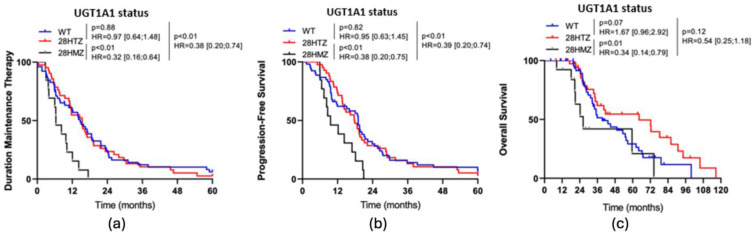
(**a**) Kaplan-Meier curve of the DMT depending on UGT1A1 status; (**b**) Kaplan-Meier curve of PFS depending on UGT1A1 status; (**c**) Kaplan-Meier curve of OS depending on UGT1A1 status.

Patients with homologous mutations of UGT1A1 had a significantly lower DMT in comparison with patients with WT (HR: 0.38, 95% CI 0.20–0.74, *p* < 0.01) and heterozygous mutations (HR = 0.32, 95% CI 0.16–0.64, *p* < 0.01). PFS was also significantly lower for patients with homologous mutations of UGT1A1 in comparison with patients with WT (HR: 0.39, 95% CI 0.20–0.74, *p* < 0.01) and heterozygous mutations (HR = 0.38, 95% CI 0.20–0.75, *p* < 0.01). There was a significantly lower overall survival in patients with homologous mutations of UGT1A1 compared to patients with heterozygous mutations of UGT1A1 (HR: 0.34, 95% CI: 0.14–0.79, *p* = 0.01) (see Figure 4).

## 4. Discussion

Our study presents a «real life» cohort of metastatic colorectal cancer patients. The treatment took place between March 2014 and June 2022 and, therefore, largely represents current clinical practice and recent treatment guidelines.

Looking at the overall patient characteristics, the patient cohort had a relatively severe disease burden, with over three-quarters of the patients presenting with synchronous metastases and more than half of the patients having two metastatic sites or peritoneal disease. Baseline CEA was also high, with a mean of 418 U/L.

National and European guidelines recommend a first-line doublet or triplet chemotherapy regimen as induction therapy associated with targeted therapy depending on the KRAS, NRAS and BRAF status for patients with pMMR/MSS non-resectable metastatic colorectal cancer [3]. Approximately 45% of patients received a triplet chemotherapy regime by FOLFIRINOX as induction therapy, while 55% of patients received a doublet chemotherapy with FOLFOX (50%) or FOLFIRI (5%).

When comparing the triplet and doublet induction chemotherapy groups, the analysis shows that patients receiving triplet chemotherapy were younger and in better general condition. However, they presented a more aggressive disease with a higher rate of synchronous metastases and a higher mean baseline CEA, both known as poor prognostic factors. Patients who received induction triplet chemotherapy had a higher proportion of rectal tumours, whereas patients receiving doublet chemotherapy had a high proportion of left-sided colon tumours.

In this study, induction triplet chemotherapy did not significantly increase the objective response rate but significantly increased the CEA delta compared to doublet chemotherapy. Induction triplet chemotherapy also led to a significantly higher rate of liver surgery and a trend for a higher rate of local treatment of metastases. In our analysis, both nadir CEA and local treatment of liver and lung metastases were proven to increase the DMT. Thus, induction triplet chemotherapy, by reducing the tumour burden, gives patients access to surgical treatments, which increases the DMT and OS. DMT, PFS and OS were equivalent across the two treatment groups. However, such conclusions should be softened by the difference in clinical characteristics between the two groups.

Similar response rates between triplet and doublet chemotherapy regimens could appear as a surprising result. However, doublet chemotherapy associated with anti-EGFR Cetuximab in the CELIM study achieved response rates close to those of triplet chemotherapy and anti-VEGF Bevacizumab in the OLIVIA trial [24,25].

Previous studies, such as the TRIBE trial, demonstrated that triplet chemotherapy and anti-VEGF Bevacizumab are superior to doublet chemotherapy by FOLFIRI and Bevacizumab for PFS and OS [22]. However, backbone doublet chemotherapy was mainly FOLFOX in our cohort of patients, for which there is less data in comparison with triplet chemotherapy and targeted therapies. FOLFIRINOX and Bevacizumab were compared to mFOLFOX6 and Bevacizumab in the Phase II OLIVIA trial: FOLFIRINOX and Bevacizumab were associated with a higher ORR and PFS, at the price of higher toxicity [25].

Also, triplet chemotherapy and anti-VEGF have never been directly compared in a randomised study to doublet chemotherapy and anti-EGFR for RAS WT patients. Our results suggest that the use of doublet chemotherapy and targeted therapy according to the KRAS, NRAS and BRAF status is equivalent to triplet chemotherapy and an anti-VEGF agent for PFS and OS. Nonetheless, prospective trials are warranted to validate such a hypothesis.

The primary endpoint of our study was to determine factors influencing the duration of maintenance therapy (DMT), an interval of time with fewer cancer-related symptoms and fewer treatment-related toxic effects for mCRC patients. Univariate analysis showed that patient characteristics such as advanced age and poor performance status in conjunction with CRC clinical characteristics, including N2 and M1b/c stages, synchronous occurrence of metastases and higher levels of baseline CEA were associated with a shorter DMT. The induction chemotherapy regimen did not significantly influence the DMT. However, the nadir CEA was associated with DMT. Maintenance chemotherapy regimen did not significantly influence the DMT, and somewhat surprisingly, anti-VEGF maintenance targeted therapies were associated with a shorter DMT. However, surgical treatments, including primary tumour resection and local treatment of liver and lung metastases (surgery, radiotherapy, thermal ablation), significantly increased the DMT. Age, N2 and M1c status, baseline CEA and local treatment of liver metastases remained significant in multivariate analysis. These results support the fact that low initial tumour burden and surgery of the primary tumour and liver and lung metastases are the main factors that determine DMT.

In the PRODIGE 9 study, high baseline leukocyte count and the lack of CEA decrease at first evaluation were associated with early progression and short duration of maintenance treatment. Also, anti-VEGF agent Bevacizumab as maintenance therapy did not show an improvement in PFS [41]. In the PANAMA trial, the depth of response was the main determinant associated with maintenance duration [39]. Our data suggests that initial tumour burden could also be an important determinant.

In our study, the maintenance chemotherapy regimen did not significantly influence the DMT. These results contrast with those of the OPTIMOX2 study in which chemotherapy-free intervals were deleterious on PFS compared to LV5FU maintenance therapy [42]. However, several other studies, such as the COIN and the FOCUS4-N trials, have shown that intermittent chemotherapy was not deleterious on OS [35,37]. The decision of the type of maintenance therapy remains a matter of debate. Currently, our data simply suggests that systematic usage of maintenance therapy provides modest benefits, and a patient-tailored selection for a break in chemotherapy might be a safe solution to limit chemotherapy toxicity.

Surgical treatments have evolved over the past decade, with more imaging and surgical methods enabling the operation of a higher number of patients. A high proportion of patients in our cohort underwent PTR and local treatment of metastases. In the recent CAIRO4 prospective study, upfront primary tumour resection associated with systemic induction therapy did not result in a survival benefit for unselected patients with unresectable metastatic colorectal cancer [30]. However, the role of PTR during maintenance therapy has not been studied. The benefit of PTR in our study may be due to a selection bias, but it may also reduce maintenance therapy chemoresistance by decreasing tumour mass. Our data also confirms the benefit of local treatment of liver metastases in mCRC, which should be considered in all cases where and when the metastases are resectable [43].

In our cohort of patients, PTR and local treatment of liver metastases were favourable for OS in univariate analysis, and PTR remained significant in multivariate analysis.

The study of pharmacogenetic mutations of DPYD and UGT1A1 genes identified different patient subpopulations for which we compared treatment characteristics. The subgroup of patients with UGT1A1 homologous mutations was treated with a significantly reduced induction dose intensity and trend for a lower cumulated dose of Irinotecan compared to patients with WT or a heterozygous mutation of UGT1A1. This Irinotecan dose reduction is based on evidence of a higher occurrence and intensity of Irinotecan-induced toxicity, such as neutropenia and diarrhoea, in that subgroup of patients [32,33]. Our analysis showed that these patients also had a significantly lower DMT, PFS and OS. The role of a low dosage of Irinotecan in the poorer outcomes of such patients could be discussed.

The limitations of our study are its retrospective design and monocentric recruitment. The relatively small number of patients in the cohort treated during the inclusion period may be responsible for a lack of power in finding statistically significant associations. In addition, with the number of dMMR/MSI patients being lower than expected, this patient population was underrepresented. However, we studied a cohort of unselected patients, representing “real life” practice. The high OS and PFS may suggest a population bias with enrichment in patients with favourable prognostic factors. In addition, the better OS in patients not receiving maintenance chemotherapy regimens may be explained by immortal-time bias. Other limitations of our study are that it compares a heterogeneous population of patients in terms of initial patient characteristics, and patients were not randomised to treatment according to all known prognostic factors. This could account for selection bias of the treatment and the related outcome. However, the absence of difference in DMT, PFS and OS between triplet and doublet chemotherapy regimens, despite the influence of medical choice in the assignment of patients to one or another regimen, is an argument in favour of a modest difference in clinical outcome between these two strategies.

## 5. Conclusions

Duration of maintenance therapy is influenced by initial patient and colorectal cancer characteristics. In addition, the duration of maintenance therapy is significantly increased by primary tumour resection and local treatment of liver and lung metastases. By reducing tumour burden, a triplet induction chemotherapy regimen gives patients access to local treatment of metastases.

Patients with homologous mutations of UGT1A1 have a worse prognosis, potentially due to reduced administration of Irinotecan.

## Figures and Tables

**Table 1:** Patient characteristics.

Characteristics		*n* = 133 Patients (%)
Patient characteristics		
Age (years), mean (range)		65 (26–88)
Gender, n (%)	Male	79 (59)
	Female	54 (41)
Performance status, n (%)	0	57 (43)
	1	61 (46)
	2	15 (11)
Pharmacogenetic characteristics		
DPYD, n (%)	Proficient	96 (91)
	Intermediate	8 (8)
	Deficient	1 (1)
UGT1A1, n (%)	Wild type	53 (49)
	Heterozygous	42 (39)
	Homologous	13 (12)
Colorectal cancer clinical characteristics		
Primary tumour location, n (%)	Left colon	49 (37)
	Right and transverse colon	36 (27)
	Rectum	46 (35)
Tumour status ^1^, n (%)	T2	7 (6)
	T3	77 (67)
	T4	31 (27)
Nodal status ^1^, n (%)	N0	16 (17)
	N1	40 (43)
	N2	36 (39)
Metastatic status ^1^, n (%)	M1a (1 metastatic site)	63 (47)
	M1b (≥2 metastatic sites)	33 (25)
	M1c (peritoneal metastases)	37 (28)
Occurrence of metastases, n (%)	Metachronous	31 (23)
	Synchronous	102 (77)
CEA baseline (U/L), mean (range)		418 (0–14,459)
Intestinal (sub)occlusion, n (%)		29 (22)
Colorectal cancer histological and molecular characteristics		
Histological subtype, n (%)	Lieberkuhnian adenocarcinoma	127 (95)
	Mucinous adenocarcinoma	3 (2)
	Signet ring cell carcinoma	1 (1)
	Undifferentiated	2 (2)
Gene mutation, n (%)	KRAS	74 (58)
	NRAS	5 (4)
	BRAF	10 (8)
Mismatch Repair and Microsatellite instability status, n (%)	pMMR/MSS dMMR/MSI	101 (97)
		3 (3)
Treatment characteristics		
Induction chemotherapy regimen, n (%)	FOLFIRINOX	60 (45)
	FOLFOX	66 (50)
	FOLFIRI	7 (5)
Induction chemotherapy duration (months), median (range)		3.5 (2.4–4.8)
Induction targeted therapy, n (%)	Anti-EGFR ^2^	30 (22)
	Anti-VEGF ^3^	73 (55)
	None	25 (19)
Induction therapy initial radiologic response, n (%)	CR	5 (4)
	PR	98 (76)
	SD	21 (16) 5 (4)
	PD	
Induction therapy biological response	CEA nadir, (U/L), mean (range) CEA delta (%), mean (range)	29 (0–627)
		80 (42–94)
Maintenance chemotherapy regimen, n (%)	Doublet chemotherapy (FOLFOX, FOLFIRI(3), CAPOX)	31 (25)
	Single chemotherapy (LV5FU2, Capecitabine)	66 (53)
	No chemotherapy	28 (22)
Maintenance targeted therapy, n (%)	Anti-EGFR ^2^	21 (17)
	Anti-VEGF ^3^ None	62 (49)
		40 (32)
Primary tumour resection, n (%)		95 (72)
Metastases local treatment, n (%)	Liver	44 (33)
	Liver surgery	39 (29)
	Lung	17 (13)
	Peritoneum	13 (10)
	Total	70 (53)
Survival characteristics		
Maintenance treatment duration (months), median (CI)		13.9 (1.2–15.6)
1st line progression-free survival (months), median (CI)		17.9 (14.9–19.4)
Overall survival (years), median (CI)		54.9 (39.4–66.5)

DPYD: dihydropyrimidine dehydrogenase; UGT1A1: uridine diphosphate glucuronosyltransferase; CEA: carcinoembryonic antigen; pMMR/MSS—proficient mismatch repair/microsatellite stable; dMMR/MSI—deficient mismatch repair /microsatellite instability; FOLFIRINOX: 5-FU, Irinotecan, Oxaliplatin; FOLFOX: 5-FU and Oxaliplatin; FOLFIRI: 5-FU and Irinotecan; CAPOX: Capecitabine and Oxaliplatin; LV5FU2: 5-FU and Leucovorin; EGFR: epidermal growth factor receptor; VEGF: vascular endothelial growth factor; CR: complete response; PR: partial response; SD: stable disease; PD: progressive disease; ^1^: TNM AJCC 8th edition; ^2^: Anti-EGFR: Panitumumab, Cetuximab; ^3^: Anti-VEGF: Bevacizumab, Aflibercept.

**Table 2:** Comparison of the characteristics of patients treated by triplet or doublet induction chemotherapy regimen.

Characteristics		Triplet Chemotherapy n = 60	Doublet Chemotherapy n = 73	*p*-Value
Patient characteristics				
Age (years), mean (range)		61 (26–78)	68 (41–88)	**<0.001**
Performance status, n (%)	0	33 (55)	24 (33)	**0.006**
	1	25 (42)	36 (49)	
	2	2 (3)	13 (18)	
Colorectal cancer clinical characteristics				
Primary tumour location, n (%)	Left colon	18 (30)	31 (44)	**0.038**
	Right and transverse colon	14 (23)	22 (31)	
	Rectum	28 (47)	18 (25)	
Metastatic status ^1^, n (%)	M1a (1 metastatic site)	30 (50)	33 (45)	0.3
	M1b (≥2 metastatic sites)	17 (28)	16 (22)	
	M1c (peritoneal metastases)	13 (22)	24 (33)	
Occurence of metastases, n (%)	Metachronous	8 (13)	23 (32)	**0.014**
	Synchronous	52 (87)	50 (68)	
CEA baseline (U/L), mean (range)		683 (1–14,459)	185 (0–4.593)	**0.003**
Treament characteristics				
Induction chemotherapy duration (months), median (IQR)		3.3 (2.4–4.6)	3.7 (2.5–4.9)	0.3
Induction targeted therapy, n (%)	Anti-EGFR ^2^	14 (24)	16 (22)	**0.032**
	Anti-VEGF ^3^	34 (57)	39 (54)	
	None	7 (12)	18 (25)	
Induction therapy initial radiologic response, n (%)	CR	1 (2)	4 (6)	0.4
	PR	48 (81)	50 (71)	
	SD	9 (15)	12 (17)	
	PD	1 (2)	4 (6)	
Induction therapy biological response	CEA nadir, (U/L), mean (range)	39 (1–627)	20 (0–246)	0.072
	CEA delta (%),mean	70	44	**0.047**
Maintenance chemotherapy regimen, n (%)	Doublet chemotherapy (FOLFOX, FOLFIRI(3), CAPOX)	31 (53)	0 (0)	**<0.001**
	Single chemotherapy (LV5FU2, Capecitabine)	17 (19)	49 (73)	
	No chemotherapy	10 (17)	18 (27)	
Primary tumour resection, n (%)		42 (70)	53 (74)	0.6
Metastases local treatment, n (%)	Liver	25 (42)	19 (26)	0.056
	Liver surgery	24 (40)	15 (21)	**0.014**
	Lung	8 (13)	9 (12)	0.9
	Peritoneum	8 (13)	5 (7)	0.2
	Total	37 (62)	33 (45)	0.059

CEA: carcinoembryonic antigen; FOLFOX: 5-FU and Oxaliplatin; FOLFIRI: 5-FU and Irinotecan; CAPOX: Capecitabine and Oxaliplatin; LV5FU2: 5-FU and Leucovorin; EGFR: epidermal growth factor receptor; VEGF: vascular endothelial growth factor; CR: complete response; PR: partial response; SD: stable disease; PD: progressive disease; ^1^: TNM AJCC 8th edition; ^2^: Anti-EGFR: Panitumumab, Cetuximab; ^3^: Anti-VEGF: Bevacizumab, Aflibercept. Bold numbers: *p* < 0.05.

**Table 3:** Determination of the characteristics influencing the duration of maintenance therapy in univariate analysis.

Characteristics		HR (95% CI)	*p*-Value
Patient characteristics			
Age (years)		1.01 (1.00–1.03)	0.072
Performance status	0		
	1	1.48 (1.02–2.14)	**0.039**
	2	2.54 (1.36–4.74)	**0.004**
Colorectal cancer clinical characteristics			
Primary tumour location	Left colon	-	
	Right and transverse colon	1.20 (0.77–1.87)	0.4
	Rectum	1.27 (0.84–1.94)	0.3
Tumour status ^1^	T2–T3	-	
	T4	1.41 (0.93–2.15)	0.11
Nodal status ^1^	N0–N1	-	
	N2	1.91 (1.24–2.95)	**0.003**
Metastatic status ^1^	M1a (1 metastatic site)	-	
	M1b (≥2 metastatic sites)	1.71 (1.11–2.64)	**0.015**
	M1c (peritoneal metastases)	1.84 (1.20–2.83)	**0.005**
Occurrence of metastases	Metachronous	-	
	Synchronous	1.54 (1.02–2.35)	**0.042**
CEA baseline, mean		1.09 (1.01–1.18)	**0.026**
Colorectal cancer histological and molecular characteristics			
Gene mutation	KRAS	1.13 (0.75–1.69)	0.6
	NRAS	1.85 (0.67–5.10)	0.2
	BRAF	1.29 (0.59–2.79)	0.5
Mismatch Repair and Microsatellite instability status	pMMR/MSS dMMR/MSI		
		0.32 (0.08–1.35)	0.12
Treatment characteristics			
Induction chemotherapy regimen	Doublet chemotherapy (FOLFOX, FOLFIRI)	-	
	Triplet chemotherapy (FOLFIRINOX)	0.95 (0.66–1.36)	0.8
Induction targeted therapy	None	-	
	Anti-EGFR ^2^	1.47 (0.86–2.52)	0.2
	Anti-VEGF ^3^	1.33 (0.84–2.11)	0.2
Induction therapy initial radiologic response	PD/SD	-	
	PR/CR	0.70 (0.45–1.10)	0.12
Induction therapy biological response	CEA nadir (mean, range) CEA delta (%) (mean, range)	1.21 (1.08–1.35)	**0.001**
		1.07 (0.81–1.41)	0.6
Maintenance chemotherapy regimen	No chemotherapy	-	
	Single chemotherapy (LV5FU2, Capecitabine)	1.40 (0.87–2.27)	0.2
	Doublet chemotherapy (FOLFOX, FOLFIRI(3), CAPOX)	1.18 (0.69–2.04)	0.5
Maintenance targeted therapy	None	-	
	Anti-EGFR ^2^	1.67 (0.96–2.90)	0.068
	Anti-VEGF ^3^	1.66 (1.09–2.53)	**0.018**
Primary tumour resection		0.45 (0.30–0.68)	**<0.001**
Metastases local treatment	Liver	0.61 (0.41–0.90)	**0.012**
	Liver surgery	0.54 (0.36–0.81)	**0.003**
	Lung	0.40 (0.21–0.75)	**0.005**
	Peritoneum	0.89 (0.49–1.61)	0.7
	Total	0.54 (0.38–0.77)	**<0.001**

CEA: carcinoembryonic antigen; pMMR/MSS—proficient mismatch repair/microsatellite stable; dMMR/MSI—deficient mismatch repair /microsatellite instability; FOLFIRINOX: 5-FU, Irinotecan, Oxaliplatin; FOLFOX: 5-FU and Oxaliplatin; FOLFIRI: 5-FU and Irinotecan; CAPOX: Capecitabine and Oxaliplatin; LV5FU2: 5-FU and Leucovorin; EGFR: epidermal growth factor receptor; VEGF: vascular endothelial growth factor; CR: complete response; PR: partial response; SD: stable disease; PD: progressive disease; ^1^: TNM AJCC 8th edition; ^2^: Anti-EGFR: Panitumumab, Cetuximab; ^3^: Anti-VEGF: Bevacizumab, Aflibercept. Bold numbers: *p* < 0.05.

**Table 4:** Determination of the characteristics influencing the duration of maintenance therapy in multivariate analysis.

Characteristics		HR (95% CI)	*p*-Value
Patient characteristics			
Age (years)		1.02 (1.00–1.04)	**0.031**
Performance status	0	-	
	1	0.89 (0.54–1.49)	0.7
	2	1.56 (0.70–3.50)	0.3
Colorectal cancer clinical characteristics			
Nodal status ^1^	N0–N1	-	
	N2	1.78 (1.09–2.89)	**0.021**
Metastatic status ^1^	M1a (1 metastatic site)	-	
	M1b (≥2 metastatic sites)	1.51 (0.87–2.61)	0.14
	M1c (peritoneal metastases)	2.05 (1.25–3.36)	**0.004**
Occurrence of metastases	Metachronous	-	
	Synchronous	0.84 (0.46–1.54)	0.6
CEA baseline, mean		1.10 (1.00–1.20)	0.052
Treatment characteristics			
Induction therapy biological response	CEA nadir (mean, range)	1.05 (0.85–1.31)	0.6
Maintenance targeted therapy	None	-	
	Anti-EGFR ^2^	1.46 (0.51–4.22)	0.5
	Anti-VEGF ^3^	1.51 (0.88–2.60)	0.13
Primary tumour resection		0.70 (0.42–1.17)	0.2
Metastases local treatment	Liver	0.49 (0.28–0.86)	**0.013**
	Lung	0.84 (0.34–2.08)	0.7

CEA: carcinoembryonic antigen; EGFR: epidermal growth factor receptor; VEGF: vascular endothelial growth factor ^1^: TNM AJCC 8th edition; ^2^: Anti-EGFR: Panitumumab, Cetuximab; ^3^: Anti-VEGF: Bevacizumab, Aflibercept. Bold numbers: *p* < 0.05.

**Table 5:** Comparison of the characteristics of pharmacogenetic subpopulations based on DPYD polymorphism.

Characteristics		DPYD Proficient n = 95	DPYD Intermediate or Deficient n = 9	*p*-Value
Treament Characteristics				
Induction chemotherapy, n (%)	FOLFIRINOX	48 (51)	3 (33)	0.3
	FOLFOX	42 (44)	5 (56)	
	FOLFIRI	5 (5)	1 (11)	
Induction chemotherapy 5-FU Dose intensity (%), mean		94	89	0.5
Induction therapy initial radiologic response, n (%)	CR	3 (3)	0 (0)	0.6
	PR	69 (74)	5 (62)	
	SD	16 (17)	3 (38)	
	PD	5 (5)	0 (0)	
Maintenance chemotherapy regimen, n (%)	Doublet chemotherapy (FOLFOX, FOLFIRI(3), CAPOX)	23 (26)	2 (22)	>0.9
	Single chemotherapy (LV5FU2, Capecitabine)	46 (52)	5 (56)	
	No chemotherapy	19 (22)	2 (22)	
5 FU cumulated dose (mg), mean		114,925	126,456	>0.9

DPYD: dihydropyrimidine dehydrogenase; FOLFIRINOX: 5-FU, Irinotecan, Oxaliplatin; FOLFOX: 5-FU and Oxaliplatin; FOLFIRI: 5-FU and Irinotecan; CAPOX: Capecitabine and Oxaliplatin; LV5FU2: 5-FU and Leucovorin; CR: complete response; PR: partial response; SD: stable disease; PD: progressive disease.

**Table 6:** Comparison of the characteristics of pharmacogenetic subpopulations based on UGT1A1 polymorphism.

Characteristics		UGT1A1 Wild Type n = 53	UGT1A1 Heterozygous n = 41	UGT1A1 Homologous n = 13	*p*-Value
Treament Characteristics					
Induction chemotherapy, n (%)	FOLFIRINOX	29 (55)	24 (59)	2 (15)	0.059
	FOLFOX	21 (40)	15 (37)	10 (77)	
	FOLFIRI	3 (6)	2 (5)	1 (8)	
Induction chemotherapy Irinotecan Dose intensity (%), mean		86	83	37	**0.025**
Induction therapy initial radiologic response, n (%)	CR	1 (2)	2 (5)	0 (0)	0.5
	PR	36 (71)	32 (80)	10 (77)	
	SD	10 (20)	6 (15)	2 (23)	
	PD	4 (8)	0 (0)	0 (0)	
Maintenance chemotherapy regimen, n (%)	Doublet chemotherapy (FOLFOX, FOLFIRI(3), CAPOX)	14 (29)	12 (30)	2 (15)	0.8
	Single chemotherapy (LV5FU2, Capecitabine)	23 (48)	20 (50)	9 (69)	
	No chemotherapy	11 (23)	8 (20)	2 (15)	
Irinotecan cumulated dose (mg), mean		2.932	3.290	1.556	0.13

UGT1A1: uridine diphosphate glucuronosyltransferase; FOLFIRINOX: 5-FU, Irinotecan, Oxaliplatin; FOLFOX: 5-FU and Oxaliplatin; FOLFIRI: 5-FU and Irinotecan; CAPOX: Capecitabine and Oxaliplatin; LV5FU2: 5-FU and Leucovorin; CR: complete response; PR: partial response; SD: stable disease; PD: progressive disease. Bold numbers: *p* < 0.05.

## Data Availability

Data is available on request due to ethical reasons.

## Supplementary Materials

The following supporting information can be downloaded at: https://www.mdpi.com/article/10.3390/cancers17010088/s1, Table S1: Comparison of the characteristics of patients treated by doublet, single or no maintenance chemotherapy regimen; Table S2: Determination of the characteristics influencing the overall survival in univariate analysis; Table S3: Determination of the characteristics influencing the overall survival in multivariate analysis.
