# Supplementary material for: Factors Influencing the Duration of Maintenance Therapy in Metastatic Colorectal Cancer
Source: Cancers (Basel). 2024 Dec 30;17(1):88. doi: 10.3390/cancers17010088 (PMC11720154; doi:10.3390/cancers17010088)
Supplement: Supplementary file 1 [file cancers-17-00088-s001.zip › cancers-3291510-supplementary.pdf]

Supplementary Materials

Table S1. Comparison of the characteristics of patients treated by doublet, single or no maintenance chemotherapy regimen.

Characteristics		Doublet chemotherapy n= 31[4]	Single chemotherapy n= 66[5]	No chemotherapy n=28	p-value
**Patient characteristics**					
Age (years), mean (range)		60 (56-69)	70 (60-74)	69 (61-73)	**0.025**
Performance status, n(%)	0	16 (52)	24 (36)	15 (54)	0.3
	1	13 (42)	32 (48)	12 (43)	
	2	2 (6)	10 (15)	1 (3)	
**Colorectal cancer clinical characteristics**					
Primary tumour location, n(%)	Left colon	12 (39)	22 (34)	12 (43)	0.5
	Right and transverse colon	7 (23)	22 (34)	5 (18)	
	Rectum	12 (39)	20 (31)	11 (39)	
Metastatic status[1], n(%)	M1a (1 metastatic site)	16 (52)	28 (42)	16 (57)	0.6
	M1b (≥ 2 metastatic sites)	8 (26)	15 (23)	6 (21)	
	M1c (peritoneal metastases)	7 (23)	23 (35)	6 (21)	
Occurence of metastases, n (%)	Metachronous	3 (10)	16 (24)	11 (39)	**0.029**
	Synchronous	28 (90)	50 (76)	17 (61)	
CEA baseline, (U/L), mean (range)		766 (1-14,459)	290 (0-4,593)	485 (1-10,120)	**0.068**
**Treament characteristics**					
Induction chemotherapy regimen, n(%)	FOLFIRINOX	31 (100)	17 (26)	10 (36)	**<0.001**
	FOLFOX	0 (0)	46 (70)	14 (50)	
	FOLFIRI	0 (0)	3 (4)	4 (14)	
Induction chemotherapy duration (months), median (IQR)		3.3 (2.5-4.8)	3.5 (2.4-4.5)	3.7 (2.5-5.6)	0.7
Induction targeted therapy, n (%)	Anti-EGFR[2]	10 (33)	11 (18)	7 (27)	0.13
	Anti-VEGF[3]	16 (53)	43 (67)	10 (38)	
	None	4 (13)	10 (16)	9 (35)	
Induction therapy initial radiologic response, n (%)	CR	0 (0)	3 (5)	2 (8)	0.9
	PR	25 (83)	48 (77)	19 (76)	
	SD	5 (17)	10 (16)	6 (16)	
Induction therapy biological response	CEA nadir, (U/L), mean (range)	38 (1-627)	29 (0-246)	26 (1-343)	0.6
	CEA delta (%),mean	88 (74-97)	80 (42-92)	75 (18-93)	0.13
Maintenance targeted therapy, n (%)	Anti-EGFR[2]	6 (19)	13 (20)	2 (7)	**<0.001**
	Anti-VEGF[3]	18 (58)	44 (66)	0 (0)	
	None	6 (19)	9 (14)	25 (89)	
Primary tumour resection, n (%)		23 (74)	46 (70)	22 (79)	0.7
Metastases local treatment, n(%)	Liver	15 (48)	16 (24)	13 (46)	**0.025**
	Liver surgery	15 (48)	14 (21)	10 (36)	**0.022**
	Lung	4 (13)	6 (9)	7 (25)	0.14
	Peritoneum	4 (13)	6 (9)	3 (11)	0.9
	Total	20 (65)	29 (44)	21 (75)	**0.012**

[1] :TNM AJCC 8th edition ; [2] :Anti-EGFR : Panitumumab, Cetuximab ; [3] : Anti-VEGF : Bevacizumab, Aflibercept.

Table S2. Determination of the characteristics influencing the overall survival in univariate analysis.

Characteristics		HR (95% CI)	p-value
**Patient characteristics**			
Age (years)		1.00 (0.98-1.02)	0.9
Performance status	0	-	
	1	0.97 (0.60-1.58)	>0.9
	2	3.13 (1.26-7.79)	**0.014**
**Colorectal cancer clinical characteristics**			
Primary tumour location	Left colon	-	
	Right and transverse colon	1.43 (0.82-2.49)	0.2
	Rectum	1.31 (0.76-2.24)	0.3
Tumour status[1]	T2- T3	-	
	T4	0.75 (0.43-1.32)	0.3
Nodal status[1]	N0 – N1	-	
	N2	0.67 (0.38-1.20)	0.2
Metastatic status[1]	M1a (1 metastatic site)	-	
	M1b (≥ 2 metastatic sites)	0.95 (0.52-1.72)	0.9
	M1c (peritoneal metastases)	1.68 (0.97-2.92)	**0.065**
Occurrence of metastases	Metachronous	-	
	Synchronous	0.97 (0.59-1.58)	0.9
CEA baseline (mean, range)		0.98 (0.88-1.09)	0.7
**Colorectal cancer histological and molecular characteristics**			
Gene mutation	KRAS	1.19 (0.72-1.98)	0.5
	NRAS	2.24 (0.69-7.30)	0.2
	BRAF	0.60 (0.19-1.93)	0.4
Mismatch Repair and Microsatellite instability status	pMMR/MSS	-	
	dMMR/MSI	0.14 (0.02-1.01)	**0.052**
**Treament characteristics**			
Induction chemotherapy regimen	Doublet chemotherapy (FOLFOX, FOLFIRI)	-	
	Triplet chemotherapy (FOLFIRINOX)	1.28 (0.80-2.02)	0.3
Induction targeted therapy	None	-	
	Anti-EGFR	1.64 (0.84-3.20)	0.15
	Anti-VEGF	1.58 (0.88-2.86)	0.13
Induction therapy initial radiologic response	PD /SD	-	
	PR/ CR	0.93 (0.53-1.65)	0.8
Induction therapy biological response	CEA nadir (mean, range)	1.02 (0.87-1.20)	0.8
	CEA delta (%) (mean, range)	1.00 (0.99-1.00)	0.2
Maintenance chemotherapy regimen	No chemotherapy	-	
	Single chemotherapy (LV5FU2, Capecitabine)	1.97 (1.04-3.72)	**0.037**
	Doublet chemotherapy (FOLFOX, FOLFIRI(3), CAPOX)	1.52 (0.78-3.00)	0.2
Maintenance targeted therapy	None	-	
	Anti-EGFR[2]	1.31 (0.67-2.56)	0.4
	Anti-VEGF[3]	1.21 (0.72-2.04)	0.5
Primary tumour resection		0.52 (0.28-0.94)	**0.032**
Metastases local treatment	Liver	0.60 (0.38-0.97)	**0.036**
	Liver surgery	0.69 (0.44-1.08)	0.10

Lung	0.97 (0.30-3.10)	>0.9
Peritoneum	3.46 (1.75-6.85)	**<0.001**
Total	1.00 (0.62-1.59)	>0.9

[1] :TNM AJCC 8th edition ; [2] :Anti-EGFR : Panitumumab, Cetuximab ; [3] : Anti-VEGF : Bevacizumab, Aflibercept.

Table S3. Determination of the characteristics influencing the overall survival in multivariate analysis.

**Characteristics**		**HR (95% CI)**	**p-value**
**Patient characteristics**			
Performance status	0	-	
	1	0.72 (0.39-1.35)	0.3
	2	2.17 (0.77-6.13)	0.14
**Colorectal cancer clinical characteristics**			
Metastatic status[1]	M1a (1 metastatic site)	-	
	M1b (≥ 2 metastatic sites)	0.71 (0.34-1.49)	0.4
	M1c (peritoneal metastases)	1.54 (0.82-2.89)	0.2
**Colorectal cancer histological and molecular characteristics**			
Mismatch Repair and Microsatellite instability status	pMMR/MSS	-	
	dMMR/MSI	0.16 (0.02-1.30)	0.086
**Treament characteristics**			
Maintenance chemotherapy regimen	No chemotherapy	-	
	Single chemotherapy (LV5FU2, Capecitabine)	1.25 (0.60-2.57)	0.5
	Doublet chemotherapy (FOLFOX, FOLFIRI(3), CAPOX)	0.72 (0.32-1.62)	0.4
Primary tumour resection		0.48 (0.23-0.99)	**0.045**
Metastases local treatment	Liver	0.69 (0.37-1.28)	0.2
	Peritoneum	3.86 (1.71-8.68)	**0.001**

[1] :TNM AJCC 8th edition.
